# Declining seroprevalence of hepatitis A in Vojvodina, Serbia

**DOI:** 10.1371/journal.pone.0217176

**Published:** 2019-06-04

**Authors:** Snežana Medić, Cleo Anastassopoulou, Vesna Milošević, Nataša Dragnić, Smiljana Rajčević, Mioljub Ristić, Vladimir Petrović

**Affiliations:** 1 Center for Disease Control and Prevention, Institute of Public Health of Vojvodina, Novi Sad, Serbia; 2 Faculty of Medicine, University of Novi Sad, Novi Sad, Serbia; 3 Division of Genetics, Cell and Developmental Biology, Department of Biology, University of Patras, Patras, Greece; 4 Center for Virology, Institute of Public Health of Vojvodina, Novi Sad, Serbia; 5 Center for Informatics and Biostatistics, Institute of Public Health of Vojvodina, Novi Sad, Serbia; CEA, FRANCE

## Abstract

To assess the current hepatitis A virus (HAV) endemicity in the Autonomous Province of Vojvodina, Serbia, we examined the seroprevalence and susceptibility profiles of the general population. A serum bank of 3466 residual samples, collected in 2015–16 as per the specifications of the European Sero-Epidemiology Network 2 project (ESEN2), was tested for anti-HAV antibodies with an enzyme immunoassay. Relationships between anti-HAV positivity and demographic features of respondents were examined by univariable and multivariable analyses. Present-day HAV seroprevalence was compared with that obtained in 1978–79. Surveillance data for hepatitis A recorded between 2008 and 2017 were also analyzed. Age was the only demographic variable found to be independently associated with a HAV seropositive status. Seropositivity (17% overall *vs*. 79% in 1978–79) increased with age to a maximum of 90% in the elderly ≥60 years. Only 5% of subjects <30 years were seropositive, unlike the 44% of seropositives ≥30 years. The estimated age at midpoint of population immunity (AMPI) increased markedly from 14 years in the late 70s to 55 years in 2015–16. Meanwhile, disease incidence decreased noticeably in recent years (from 11 in 2008 to 2 per 100,000 population in 2017). In the ongoing pre-vaccine era, natural infection provides immunity for merely a third (31%) and two thirds (57%) of people in their 40s and 50s, respectively. Hence, the majority of people ≤40 years (94%) and middle-aged adults 40–49 years (69%) are susceptible to HAV. Older susceptible individuals, particularly those ≥50 years (24%), are prone to severe symptoms. Taken together, these changes reflect the epidemiological transition of Vojvodina and Serbia from high to very low HAV endemicity, thereby supporting the current national policy of immunization of only high-risk groups.

## Introduction

Hepatitis A is an acute self-limited infectious disease caused by hepatitis A virus (HAV) that is mainly transmitted via the fecal/oral route, through exposure to contaminated food and water or direct contact with infected persons [1]. The clinical course of HAV infection is age-dependent and ranges from asymptomatic (commonly in children aged ≤5 years) to acute symptomatic hepatitis, with the higher severity of disease in older population. Fulminant hepatitis occurs in 0.015–0.5% of cases, while the estimated case-fatality ratio ranges from 0.1 in children to 1.8–5.4% in adults ≥50 years of age [2]. Despite the availability of a safe and effective vaccine, hepatitis A is the most common form of acute viral hepatitis in the world [1,3]. The World Health Organization (WHO) recommends universal childhood immunization in countries with intermediate HAV endemicity and immunization of high-risk groups in countries with low and very low endemicity [1,4].

Assessment of hepatitis A endemicity is based primarily on estimated seroprevalence of anti-HAV antibodies acquired after past infection [1]. Based on seroprevalence data, the unique national HAV seroprofiles can be determined. Endemicity levels were hence found to vary geographically, with the opposite gradient in susceptibility profiles: low-income regions were characterized by high endemicity levels, while high-income regions had very low or low HAV endemicity levels and enlarging cohorts of susceptible older adults prone to symptomatic disease and complications, paradoxically leading to increased disease burden [3,5–7]. Targeted immunization against hepatitis A for high-risk groups is currently recommended in the majority of European Union and European Economic Area (EU/EEA) countries where HAV endemicity levels are low or very low due to improved hygienic conditions, housing and sanitation [6,8]. Serosurveys conducted over a decade ago revealed low level of HAV transmission and increased susceptibility of children and young adults in most of Europe [9]. Still, recent seroprevalence data are lacking for many European countries [10,11].

Located at the crossroads of Central and Southeast Europe, the Republic of Serbia is an upper-middle-income economy country without implemented vaccination against hepatitis A. Until 2019, the hepatitis A vaccine was recommended only for international travelers to highly endemic countries. Therefore, it is reasonable to assume that most, if not all, HAV infections up to that time have been naturally acquired. Scarce HAV serosurveys carried out in small numbers of respondents in the past century had shown high overall anti-HAV seroprevalence [12]. In the Automonous Province of Vojvodina in the north of Serbia (population of ~2 million, 27% of Serbian population) [13,14], HAV-specific seroprevalence was obtained decades ago by two serosurveys [15,16]. The first study established an overall anti-HAV seroprevalence of 78.9% by testing sera from 1000 subjects in 1978–79. Seroprevalence increased with age, reaching almost complete immunity to HAV (98.7%) at the age of 30 [15]. The 1993 survey showed a lower overall anti-HAV prevalence (56.9%) and a shift in HAV seropositivity towards older age groups [16].

Herein, we present an epidemiological assessment of HAV infection in Vojvodina, Serbia by describing the present-day HAV seroprevalence and susceptibility in the general population of the province in comparison to data dating back from 1978–79. Analysis of recent surveillance data for hepatitis A in conjunction with HAV seroprevalence and susceptibility provides a rationale that is expected to contribute to the evaluation and potential adjustment of the existing national immunization strategy.

## Materials and methods

### Survey design

The Institute of Public Health of Vojvodina (IPHV) conducted a serosurvey between April 2015 and March 2016 in order to assess the level of immunity of the local population against vaccine-preventable diseases. For the purposes of this survey, a main bank consisting of residual diagnostic sera was collected mostly from urban areas of three principal geographic zones of the province: Northern, Central, and Southern Vojvodina. The study protocol for the collection of sera followed the specifications of the European Sero-Epidemiology Network 2 (ESEN2) project [9], as previously described [17]. The age, gender and geographic stratification of the serum bank were representative for the population of Vojvodina as per the latest census [13]. HAV seropositivity was thus determined on sera obtained from 3466 subjects of all ages and with an equal distribution by gender (1732 males/1734 females, age range: 1–84 years; mean age±SD: 23.5±18.9 years). Samples from infants <1 year of age, immunocompromised individuals and recent recipients of blood and blood products were not included in this survey. The study was approved by the Medical Ethics Committee of IPHV and all health-care centers involved in sera collection. Written informed consent was obtained from study participants, or their parents or legal guardians if they were <15 years old. All procedures contributing to this work comply with the ethical standards of the relevant national and institutional committees on human experimentation and with the Helsinki Declaration of 1975, as revised in 2008.

### Serological assay

Sera were stored at -20°C until testing for total anti-HAV using the ADVIA Centaur HAV Total Assay (Bayer, Tarrytown, NY, USA) on the corresponding (ADVIA Centaur) system at the Virology laboratory of IPHV. The assay that serves as an aid in the diagnosis of previous or ongoing hepatitis A viral infection or, conversely, in the identification of HAV-susceptible individuals was performed and interpreted according to the manufacturer’s instructions. The clinical sensitivity and specificity of the test were reported by the manufacturer to be comparable to other current commercially available licensed assays.

### Comparison with previous studies of anti-HAV prevalence

HAV seroprevalence obtained from this survey was compared to the only available corresponding data from the previous study that had been conducted in Vojvodina in 1978–79 [15]. Seroprofiles from 1978–79 and 2015–16 were thus analyzed in parallel. For the purposes of this comparison, seroprevalence data of infants <1 year of age from the 1978–79 serosurvey were excluded from analysis to match the dataset of this (2015–16) serosurvey. The estimated age at which half of the population was HAV-seropositive, and therefore presumably immune to HAV, was determined in each case. To calculate the estimated age with 50% detectable anti-HAV, or the youngest age at midpoint of population immunity (AMPI) [18,19] as explained above, points corresponding to the seroprevalence rate identified in each age group were plotted with the midpoint of the age group on the x-axis and the prevalence rate on the y-axis. Logarithmic, polynomial and sigmoidal curves were fit to these points. The curve with r^2^ nearest to 1 was selected as the best-fit curve. We then used the equation for quadratic curve as the best-fit curve to calculate the youngest age at which seroprevalence was equal to 50%. Estimated HAV endemicity levels are considered as very high for an AMPI of <5 years, high for an AMPI of 5–14 years, intermediate for an AMPI of 15–34 years, and low for an AMPI of ≥35 years of age [18–20].

### Incidence and outbreaks of hepatitis A in Vojvodina

Notification records of all hepatitis A cases as well as data related to outbreaks reported in Vojvodina between 2008 and 2017 were extracted from the surveillance database of IPHV. The final classification as probable or confirmed hepatitis A cases was done according to the EU case definition criteria [21]. Annual crude incidence (per 100,000 individuals) of hepatitis A was calculated using the latest census data for the population of Vojvodina as a denominator [13]. The identities of all recorded HAV cases remained confidential.

### Statistical analysis

Associations between anti-HAV seroprevalence and demographic characteristics of respondents (age, gender and area of residence) were examined by the chi-square test. Multivariable logistic regression was used to determine independent associations between the aforementioned sociodemographic features and HAV infection. The expected number of seropositive inhabitants of Vojvodina was extrapolated from obtained anti-HAV seropositivity rates in the tested sera and the estimated population of Vojvodina in 2015 [14]. Data were analyzed using the IBM SPSS Statistics 21.

## Results

### HAV seroprevalence and associations with demographics

Overall, 589 of 3466 (17.0%, 95% CI: 15.8–18.3) sera tested positive, whilst 2877 (83.0%, 95% CI: 81.7–84.2) tested negative for anti-HAV antibodies. No significant associations were found between HAV seropositivity and gender or area of residence in Vojvodina (Table 1).

**Table 1:** Factors associated with anti-HAV seropositivity in Vojvodina, Serbia, 2015–16.

Factor	Population^a^ (n)	No. of tested samples	% anti-HAV seropositivity	95% CI	*P*^b^	Adjusted Odds Ratio (OR)^c^	95% CI	*P*^d^	Estimated anti-HAV seropositives (n)
**Gender**					0.568			0.39	
Male	912,306	1,732	16.6	14.9–18.5		Reference			151,442
Female	961,902	1,734	17.4	15.6–19.2		1.1	0.9–1.4		167,370
**Area of residence**					0.769			0.30	
Northern	499,068	924	17.2	14.9–19.8		1.0	0.8–1.4	0.86	85,840
Central	790,234	1,463	17.4	15.5–19.4		1.2	0.9–1.6	0.16	137,500
Southern	584,906	1,079	16.3	14.2–18.6		Reference			95,340
**Age group (years)**					**<0.001**			**<0.0001**	
1–4	70,502	400	6.8	4.6–9.7		2.2	1.2–4.2	0.01	4,794
5–9	90,518	512	3.1	1.9–5.1		Reference			2,806
10–14	95,159	512	4.1	2.7–6.2		1.3	0.7–2.6	0.40	3,902
15–19	96,996	577	5.4	3.8–7.5		1.8	0.9–3.3	0.07	5,238
20–24	112,081	201	6.0	3.3–10.2		2.0	0.9–4.2	0.08	6,725
25–29	122,893	199	8.0	4.9–12.7		2.7	1.3–5.5	0.006	9,831
30–34	132,755	200	9.0	5.7–13.8		3.1	1.5–6.1	0.002	11,948
35–39	135,428	199	17.1	12.5–23.0		6.4	3.4–11.9	<0.0001	23,158
40–49	256,237	201	30.8	24.9–37.6		13.5	11.4–30.0	<0.0001	78,921
50–59	278,259	197	56.9	49.9–63.6		41.4	23.3–73.3	<0.0001	158,329
≥60	483,380	268	89.6	85.3–92.7		269.5	142.9–508.0	<0.0001	433,108
**Total**	**1,874,208**	**3,466**	**17.0**	**15.8–18.3**					**738,760 (39.4%)**^e^

^a^ Estimated population ≥1 year of age in 2015 [14].

^b^ Chi square test

^c^ Multivariable logistic regression

^d^ Wald test.

^e^ Calculated from estimated age-specific seropositives (total) divided by the population ≥1 year of age in 2015.

Anti-HAV seroprevalence increased with age, ranging from 3.1% (95% CI: 1.9–5.1) in the 5–9 to 89.6% (95% CI: 85.3–92.7) in the ≥60 years age group. By the age of 30 years, 5.1% of subjects were seropositive, unlike the 43.7% of seropositives ≥30 years.

Multivariable logistic regression analysis showed that the age of respondents was the only demographic variable that was independently associated with a HAV seropositive status (Table 1). The adjusted odds ratio (OR) for anti-HAV seropositivity ranged from 1.3 (95% CI: 0.7–2.6) for children aged 10–14 years to 269.5 (95% CI: 142.9–508.0) for adults aged ≥60 years compared to 5-9-year-old children. The mean age of anti-HAV seropositives (48.1 ± 21.1 years) and seronegatives (18.4 ± 13.7) differed significantly (*P*<0.001).

The expected number of seropositive inhabitants of Vojvodina in 2015 extrapolated from obtained age-specific anti-HAV seropositivity rates and estimated population size is 738,760, corresponding to an overall seroprevalence of 39.4% (95% CI: 39.3–39.5) in the population ≥1 year (Table 1). By the age of 30 years, 5.7% of individuals are anticipated to be immune to HAV compared to 54.8% of people ≥30 years.

### HAV susceptibility

The vast majority of children and young adults aged ≤34 years remain susceptible to HAV (S1 Table). From the age of 35 onwards, the proportion of seronegative respondents gradually declined from 83% in the 35–39 to 69% in the 40–49 to 43% in the 50–59 years age group, to reach a minimum of 10% among those aged ≥60 years. The overall proportions of anti-HAV seronegative respondents did not differ by gender [83.4% (95% CI: 81.5–85.1) in males *vs*. 82.6% (95% CI: 80.8–84.4) in females].

### Trends of anti-HAV positivity from 1978–79 to 2015–16

**Fig 1:**
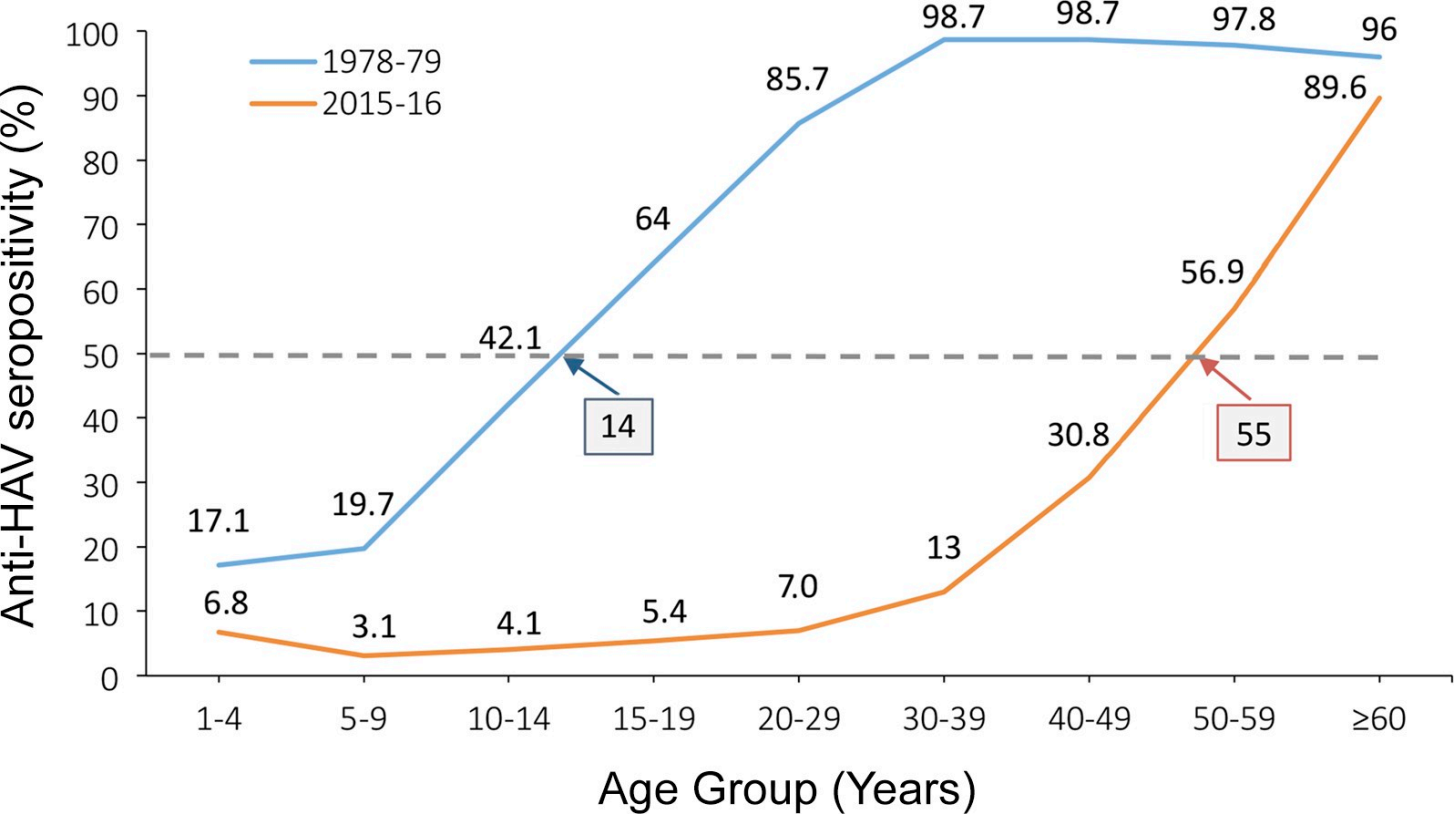
Comparison of anti-HAV positivity (%) in Vojvodina, Serbia from 1978–79 to 2015–16 (blue and reddish brown lines, respectively). The intermittent line denotes 50% anti-HAV positivity, while the points of intersection with the seroprevalence curves indicate the estimated age (years) at midpoint of population immunity (AMPI) in each time period (shown in boxes).

A comparison of the current age-specific seroprevalence with that obtained previously is presented in Fig 1.

In 1978–79, ~90% of young adults were HAV-seropositive. Almost four decades later, the seroprofile changed substantially: ~90% of young adults (93% and 87% for the 20–29 and 30–39 age groups, respectively) and ~70% (69.2%) of middle-aged adults 40–49 years are currently susceptible to HAV in Vojvodina. The prominent shift in HAV seropositivity towards older age groups over the past 37 years is best shown by estimated AMPI that progressively increased from 14 years in 1978–79 to 55 years in 2015–16 (low endemicity).

### Incidence and outbreaks of hepatitis A in Vojvodina

A total of 765 patients and 28 outbreaks of hepatitis A were notified in Vojvodina between 2008 and 2017 (S2 Table). Downward trends characterized disease incidence during these years, with average rates of 4.0/100,000 (range 0.9–11.2/100,000). Hepatitis A incidence dropped below 5.0/100,000 in the last eight years in Vojvodina (average 2.5/100,000). Outbreaks of HAV also decreased in frequency but occurred every year, principally afflicting people living under poor hygienic conditions, particularly in Roma settlements, but also children in pre-school and school facilities, hospital patients and the general population. No deaths were reported due to hepatitis A in the observed period.

## Discussion

To our knowledge, this is the first population-based study that allows for a comprehensive assessment of the current HAV endemicity level in Vojvodina, Serbia based on the estimation of age-specific HAV seroprevalence. We found that by the age of 30 years, only 5% of subjects possessed antibodies to HAV unlike the 44% of individuals ≥30 years of age. Hence, the majority of middle-aged adults are susceptible to HAV and potentially prone to symptomatic disease, especially for those ≥50 years. A similar pattern was uncovered in Northern, Western and Central Europe [6,9], with acquisition of antibodies primarily in mid-adulthood accompanied by high or very high susceptibility of children, young and middle-aged adults. This finding constitutes the so-called paradox of HAV epidemiology since the amelioration of socio-economic and hygienic conditions and the consequent transition to low or very low endemicity leaves an enlarging cohort of susceptible older adults running the risk of symptomatic infection [2].

The lower seroprevalence detected in 2-year-olds (5%) compared to infants aged 1 year (15%) is due to the waning of maternal anti-HAV antibodies [5]. Recent longitudinal studies in infants have shown that anti-HAV IgG antibodies may persist well in the second year of life, depending on maternal anti-HAV titers [22]. By 5 years of age, 6.8% of pre-school children were seropositive similarly to the majority of EU/EEA countries in the pre-vaccine era [9]. Only 8% of 18/19-year-olds were found to be seropositive and this proportion was essentially maintained until the age of 34. Comparable low seroprevalence (4.8–9.1%) in persons <30 years of age and high HAV susceptibility (59.4% overall) was obtained in neighboring Croatia [23].

The noticeable shift of AMPI to later adulthood in Vojvodina is another important indicator of the transitioning from high to low HAV endemicity marked by the high proportion of susceptible individuals and increased risk of outbreaks in case of reintroduction of virus circulation in the community [19]. Indeed, it has been recently estimated that HAV was responsible for 14 million foodborne illnesses globally in 2010 [2]. Multi-state hepatitis A outbreaks in high-income countries are commonly related to consumption of contaminated food [24,25], sex behavior (i.e. epidemics among men-who-have-sex-with men, MSM) [26], or international travel to highly endemic regions [27]. Thus, the risk of re-establishing HAV circulation by importation of food, travel and migration in today’s globalizing world is high [28], as also reflected by the undisrupted notification of hepatitis A in EU/EEA (average incidence 2.5, range 0–26.0/100,000 population in 2015), with the majority of cases (41.3%) reported in Romania and Bulgaria [29].

The incidence of hepatitis A in Serbia has decreased steadily (from 27.7 in 2008 to 1.1 per 100,000 in 2017), averaging 1.7 per 100,000 during the last five years [30]. In the period 1988–2009, hepatitis A was maintained endemically in Vojvodina, with an average incidence of 23.4/100,000 population [31]. Community-wide outbreaks were replaced with small-scale epidemics in communities with poor sanitation followed by a further decline in disease incidence (from 11.2 in 2008 to 2.1 per 100,000 population in 2017) [32]. Of note is that HAV was not found in any of analyzed surface waters or urban sewage samples in Vojvodina [33]. This is important since it is known that HAV can persist on environmental surfaces, food and sewage for long periods of time [2].

The major strengths of this study stem directly from its design pertaining to the size (3466 sera samples tantamount to 0.2% of the population of Vojvodina) and stratification of collected sera, which allow for the generalization of results to the whole country. On the other hand, the possibility of insufficient national representativeness cannot be excluded, despite the highly similar demographic and socio-economic parameters of Vojvodina and the rest of the country. Therefore, additional serosurveys, on both the national and sub-national level, are deemed necessary to inform vaccination policy decisions against HAV. Furthermore, the ESEN2-based age stratification protocol that we followed may have been suboptimal for a more detailed assessment of anti–HAV seroprevalence in older age groups. Yet, this potential problem was alleviated to an extent by the extrapolation of the number of seropositive individuals by age groups from obtained anti-HAV seropositivity rates and estimated population size of the province. Although the serum banks and assays used in surveys from 1978–79 and 2015–16 obviously differed in many aspects, the comparison of respective seroprevalence rates is meaningful and it allowed for the detection of such gross effects as the epidemiological shift of HAV infections.

The new national legislation recommends vaccination against HAV for high-risk groups aged ≥16 years, starting from 2019 [34,35]. The immunization policy mandates vaccination for the following high-risk groups: intravenous drug users (IDVUs), liver transplantation patients, persons working under poor hygienic conditions (e.g. in waste disposal and cemeteries) and homosexual men. The new law that will take effect starting from 2019 further introduces recommendations for vaccination of non-immune individuals without any special risk.

In conclusion, this study provides a comprehensive assessment of the HAV endemicity transition of Vojvodina and Serbia over time and evidences that HAV circulation has gradually declined over the past four decades. Hence, Serbia may be classified as a very low endemicity country nowadays, supporting the national vaccination policy of high-risk groups. The presented evidence of occasional local HAV circulation and apparent risk of transmission to the growing cohort of susceptible adults support the need to reinforce the overall prevention and control strategies for hepatitis A in Serbia.

## Supporting information

S1 Table Proportion of anti-HAV seronegative respondents by age and gender in Vojvodina, Serbia, 2015–16. (DOC)

S2 Table Annual number of reported cases, incidence and outbreaks of hepatitis A in Vojvodina, 2008–2017. (DOC)
