# Supplementary material for: Declining seroprevalence of hepatitis A in Vojvodina, Serbia
Source: PLoS One. 2019 Jun 4;14(6):e0217176. doi: 10.1371/journal.pone.0217176 (PMC6548380; doi:10.1371/journal.pone.0217176)
Supplement: S1 Table — (DOC) [file pone.0217176.s001.doc]

**S1 Table.** Proportion of anti-HAV seronegative respondents by age and gender in Vojvodina, Serbia, 2015-16.^a^

| **Age group**  **(years)** | **Males**  **(%)** | **95% CI** | **Females**  **(%)** | **95% CI** | **Total seronegative**  **(%)** | **95% CI** |
| --- | --- | --- | --- | --- | --- | --- |
| 1 | 89.8 | 77.8-96.0 | 80.4 | 67.3-89.2 | 85.0 | 76.6-90.8 |
| 2 | 94.0 | 83.2-98.6 | 96.0 | 85.8-99.7 | 95.0 | 88.5-98.1 |
| 3 | 96.0 | 85.8-99.7 | 98.0 | 88.5-100.0 | 97.0 | 912-99.3 |
| 4 | 93.8 | 82.5-98.5 | 98.1 | 88.9-100.0 | 96.0 | 89.8-98,8 |
| 5 | 100.0 | 91.5-100.0 | 95.9 | 85.5-99.6 | 98.0 | 92.5-99.9 |
| 6 | 100.0 | 91.6-100.0 | 98.1 | 88.9-100.0 | 99.0 | 94.2-100.0 |
| 7 | 96.2 | 86.5-99.7 | 98.0 | 88.5-100.0 | 97.1 | 91.4-99.4 |
| 8 | 98.0 | 88.7-100.0 | 100.0 | 91.8-100.0 | 99.0 | 94.2-100.0 |
| 9 | 88.5 | 76.7-95.0 | 94.2 | 83.8-98.6 | 91.3 | 84.2-95.6 |
| 10 | 96.0 | 85.8-99.7 | 90.0 | 78.2-96.1 | 93.0 | 86.0-96.9 |
| 11 | 96.1 | 86.0-99.7 | 96.0 | 858-99.7 | 96.0 | 89.9-98.8 |
| 12 | 98.0 | 88.5-100.0 | 96.2 | 86.5-99.7 | 97.1 | 91.4-99.4 |
| 13 | 94.2 | 83.8-98.6 | 98.1 | 88.9-100.0 | 96.2 | 90.2-98.8 |
| 14 | 98.1 | 88.9-100.0 | 96.2 | 86.3-99.7 | 97.1 | 91.5-99.4 |
| 15 | 96.4 | 87.0-99.7 | 96.5 | 87.4-99.7 | 96.4 | 90.9-98.9 |
| 16 | 96.4 | 87.2-99.7 | 100.0 | 92.3-100.0 | 98.2 | 93.3-99.9 |
| 17 | 98.3 | 90.0-100.0 | 93.1 | 83.1-97.8 | 95.7 | 90.9-98.4 |
| 18 | 91.4 | 81.0-96.7 | 93.0 | 82.8-97.7 | 92.2 | 85.6-96.0 |
| 19 | 98.4 | 90.4-100.0 | 83.6 | 72.2-91.0 | 91.0 | 84.4-95.0 |
| 20-24 | 94.1 | 87.4-97.5 | 94.0 | 87.3-97.5 | 94.0 | 898-96.7 |
| 25-29 | 90.0 | 82.4-94.7 | 93.9 | 87.1-97.5 | 92.0 | 87.3-95.1 |
| 30-34 | 92.0 | 84.8-96.1 | 90.0 | 82.4-94.7 | 91.0 | 86.2-94.3 |
| 35-39 | 82.0 | 73.2-88.4 | 83.8 | 75.2-89.9 | 82.9 | 77.0-87.5 |
| 40-49 | 71.3 | 61.8-79.2 | 67.0 | 57.3-75.5 | 69.2 | 62.4-75.1 |
| 50-59 | 45.9 | 36.4-55.8 | 40.4 | 31.3-50.3 | 43.1 | 36.4-50.1 |
| ≥60 | 9.6 | 5.6-15.9 | 11.3 | 6.8-17.9 | 10.4 | 7.3-14.7 |
| **Total** | **83.4** | **81.5-85.1** | **82.6** | **80.8-84.4** | **83.0** | **81.7-84.2** |

^a^ *P*<0.01 for differences in seroprevalence across all age groups, for each gender and in total.
