# Supplementary material for: Declining seroprevalence of hepatitis A in Vojvodina, Serbia
Source: PLoS One. 2019 Jun 4;14(6):e0217176. doi: 10.1371/journal.pone.0217176 (PMC6548380; doi:10.1371/journal.pone.0217176)
Supplement: S2 Table — (DOC) [file pone.0217176.s002.doc]

**S2 Table.** Annual number of reported cases, incidence and outbreaks of hepatitis A in Vojvodina , 2008-2017.

| **Year** | **Number of cases** | **Incidence**  **(/100,000)** | **Number of outbreaks** | **% of cases reported in outbreaks** |
| --- | --- | --- | --- | --- |
| 2008 | 216 | 11.2 | 6 | 28.2 |
| 2009 | 160 | 8.3 | 5 | 63.7 |
| 2010 | 53 | 2.7 | 3 | 41.5 |
| 2011 | 40 | 2.1 | 0 | 0 |
| 2012 | 68 | 3.5 | 6 | 63.2 |
| 2013 | 94 | 4.9 | 4 | 63.8 |
| 2014 | 44 | 2.3 | 1 | 18.2 |
| 2015 | 17 | 0.9 | 1 | 11.8 |
| 2016 | 32 | 1.7 | 1 | 9.4 |
| 2017 | 41 | 2.1 | 1 | 41.5 |
| **Total** | **765** | **NA^1^** | **28** | **NA^1^** |
| **Average** | **76.5** | **4.0** | **2.8** | **34.1** |

^1^ Not applicable.
